# Retrospective analysis on the agreement between skin prick test and serum food specific IgE antibody results in adults with suspected food allergy

**DOI:** 10.1186/s13223-016-0136-y

**Published:** 2016-07-25

**Authors:** Ling Ling, Maria B. Ospina, Kyriaki Sideri, Harissios Vliagoftis

**Affiliations:** Department of Medicine, University of Alberta, Edmonton, AB T6G 2S2 Canada; Alberta Health Services, Respiratory Health Strategic Clinical Network, Edmonton, AB T6G 2G3 Canada

**Keywords:** Adult, Food allergy, Skin prick test, Specific IgE, Agreement

## Abstract

**Background:**

Food allergy is a common clinical problem in adults. Given logistical barriers to conducting food challenges, the use of skin prick test (SPT) and specific IgE (sIgE) are important in establishing the diagnosis. The purpose of this study is to investigate the agreement of SPT and sIgE results in adults presenting to an allergy clinic with suspected food allergy.

**Methods:**

Retrospective analysis of medical records at the University of Alberta Allergy Clinic between September 2013 and May 2015 was performed. Demographic, medical history as well as SPT and specific IgE results were recorded. Agreement of SPT and sIgE for individual food allergens was analyzed by Kappa statistics.

**Results:**

Data from 260 patients was collected. The population was predominantly female, often having other atopic diseases. Very few food challenges were performed; IgE mediated food allergy was diagnosed in a minority (29.6 %) of cases. Kappa values which reached statistical significance were moderate for peanut ĸ = 0.535 (p = 0.0002, CI 0.364–0.707), walnut ĸ = 0.408 (p = 0.001 CI 0.159–0.657), pecan ĸ = 0.530 (p = 0.001 CI 0.211–0.848), and lobster ĸ = 0.543 (p = 0.004 CI 0.197–0.889), substantial for pistachio ĸ = 0.657 (p = 0.023 CI 0.224–1.000), codfish ĸ = 0.770 (p = 0.0002 CI 0.558–0.983), shrimp ĸ = 0.627 (p = 0.0006 CI 0.383–0.871) and egg white ĸ = 0.625 (p = 0.002 CI 0.293–0.957), almost perfect for cashew ĸ = 0.894 (p = 0.0008 CI 0.693–1.000) and salmon ĸ = 0.874 (p = 0.004 CI 0.705–1.000).

**Conclusions:**

The agreement between SPT and sIgE results on adults being evaluated for food allergy is at least moderate or better for peanut, walnut, pecan, pistachio, cashew, lobster, shrimp, codfish, salmon and egg white. This should be reassuring for patients who have contraindications or restricted access to either test as the results for the above allergens will likely agree. These findings may suggest that these tests could possibly be interchangeable in adults being evaluated for suspected food allergy and will aid primary care physicians in the triage of patients requiring allergist care.

## Background

Food allergy is an increasingly common diagnosis in adults. Recent prevalence estimates indicate that food allergies affect nearly 5 % of adults [1]. Oral food challenge (OFC) remains the gold standard diagnostic test for food allergy. In clinical practice, however, there are often logistical barriers to performing food challenge in outpatient settings. Lack of human resources and time are the most often listed impediments reported by allergists in an American survey [2]. As well, the possibility of inducing a systemic reaction likely weighs heavily on clinicians. Patients may resist food challenge due to fear of a reaction or are unable to make time for a prolonged visit. Due to these factors, clinicians are often relying upon skin prick test (SPT) or measurement of the antigen specific Immunoglobulin E (sIgE) to adjunct history and physical exam to make the diagnosis. The accuracy of SPT can be confounded by patients’ medication and the preparation of the allergen used in the SPT. Both SPT and sIgE can reflect cross reactivity with other allergens or asymptomatic sensitization [1]. The 2010 National Institute of Allergy and Infectious Diseases Sponsored Expert Panel Report recommends either skin prick test (SPT) or serum sIgE level as adjunctive objective testing [3]. This recommendation likely reflects the paucity of literature on the level of agreement between these two tests, despite their good performance characteristics. In Schoos et. al’s [4] examination of a birth cohort of children, agreement of SPT and sIgE to common food allergens was initially poor to moderate and deteriorated to slight agreement with age. In contrast, Asha’ari et al. [5] showed a positive correlation between SPT and sIgE in an adult population unselected for common food allergens. As these studies on the agreement of SPT and sIgE seem to have contradictory findings, further study is needed to clarify this question. The aim of this study is to investigate the level of agreement between SPT and serum sIgE results in a selected adult population referred to an academic allergy clinic for investigation of suspected food allergy. Because these two tests are independent methods of testing for sensitization, we hypothesized their results will show high levels of agreement but that the level of agreement would vary between individual food allergens.

## Methods

We performed a retrospective study/chart review of the SPT and sIgE results of adult patients seen at the University of Alberta Allergy Clinic in Edmonton (Alberta) for suspected food allergy from September 2013 to May 2015. This is an outpatient clinic affiliated with a tertiary care academic hospital. This clinic has approximately 100 new patient visits a month. Ethics approval was obtained from the University of Alberta Research Ethics Board. Electronic medical records of visits to the clinic in the selected time period were reviewed. Minimum referral age was at least 16 years with referral base of either primary care or specialist physicians. Data retrieval was performed by one author (LL).

Demographic information including age, gender were collected for all patients. Medical history variables noted were known previous diagnosis of food allergy and offending allergen, allergic rhinitis, isolated angioedema, spontaneous urticaria, atopic dermatitis, medication allergy, venom allergy, and pollen food syndrome. Details of their presenting complaint including the nature of their reaction, whether the history of the reaction was compatible with IgE mediated food allergy, and the specific food trigger of concern were extracted from the chart. The nature of the reaction was classified as cutaneous with or without angioedema, gastrointestinal, respiratory, anaphylaxis, other or unclear. The reaction was classified as other if the symptoms were not cutaneous, gastrointestinal, and respiratory. An unclear reaction was noted if the patient could not recall the details of the reaction. The history of the reaction was classified as suggestive of IgE mediated food allergy if there was a clear immediate temporal relationship between ingestion of the food and symptoms, reaction was reproducible on subsequent exposure and responded to epinephrine or antihistamines. If not all elements were noted on the chart, record made by the attending allergist of the presence of a suggestive history was also accepted. The diagnosis of IgE mediated food allergy made by the attending allergist was based on clinical impression which included a combination of factors including the history, SPT, sIgE, oral food challenge (where available) as recommended in current guidelines. The diagnosis entered by the allergist on the electronic medical record or in their consultation letter to the referring physician was used in the data abstraction. Where available, the results of skin prick test, sIgE, complete blood count, total serum IgE and other immunoglobulin quantification were also recorded.

Skin prick testing performed at the clinic used DUOTIP-TEST® (Lincoln Diagnostics Inc) and commercial extracts purchased from Omega Laboratories Ltd (Montreal, Canada). Histamine and saline were used as positive and negative controls respectively. SPT was performed by trained clinic staff under the supervision of the attending allergist. A wheal size >3 mm was considered a positive SPT result [6]. Wheal size 3 mm or smaller were considered negative.

Specific IgE testing was either ordered by the allergy clinic at the time of the visit or already available having been ordered by the referring physician. The local laboratory uses Phadia 250 Immunocap serum assay. Specific IgE titre >0.35kU/L was considered a positive result [7]. The use of 0.35kU/L or greater as the level for a positive sIgE test was chosen to give uniformity to the data analysis and was based upon the Immunocap assay’s antibody detection threshold. This detection threshold was used for every food allergen tested. For adults, there is currently no evidence to support another positive threshold value for sIgE assays.

The agreement between SPT and sIgE for individual food allergens was analyzed by using Kappa statistics. Kappa values <0 indicate poor agreement; 0 to 0.2: slight agreement; 0.21 to 0.40: fair agreement; 0.41 to 0.6: moderate agreement; 0.61 to 0.80 substantial agreement; and 0.81 to 1.00: almost perfect agreement [8]. Only patients who underwent both SPT and sIgE for a particular food allergen were included in the kappa analysis for that allergen. All data were analyzed using IBM SPSS Statistics for Macintosh (version 23.0).

## Results

A total of 260 patients were referred to the University of Alberta Adult Allergy Clinic during the study period for evaluation of a possible food allergy. These patients were typically referred after experiencing symptoms attributed to food ingestion.

Demographic information and medical history of the patient population are summarized in Table 1. The patient population was predominantly female (70.4 %), with a mean age of 38.8 years. With the exception of two patients, one 16 and one 17 years of age, the population was comprised of adults. The majority of referrals came from primary care physicians (88.8 %) and the rest from specialists, mostly respirologists. A notable portion of patients had a history of other allergic diseases, with the most common being allergic rhinitis, followed by asthma and atopic dermatitis.

**Table 1:** Demographic and clinical characteristics of patient population (N = 260)

Characteristic	N (%) or Mean ± SD
Sex	
Female	183 (70.4 %)
Male	77 (29.6 %)
Age (years)	38.8 (13.7)
Referral source	
Primary Care	231 (88.8 %)
Respirologist	7 (2.7 %)
Allergist	2 (0.8 %)
Other	10 (3.8 %)
Unknown	10 (3.8 %)
Diagnosis of atopy	
Allergic rhinitis	120 (46.2 %)
Asthma	90 (34.6 %)
Atopic dermatitis	61 (23.5 %)
Venom allergy	5 (1.9 %)
Drug allergy	42 (16.2 %)
Urticaria	60 (23.1 %)
Eczema	61 (23.5 %)
Pollen food syndrome	35 (13.5 %)

Table 2 summarizes the nature of the food reaction as well as the rate of diagnosis of food allergy in the study population. The majority of patients reported their primary symptoms as cutaneous either with or without angioedema. Gastrointestinal symptoms, unknown reaction, and anaphylaxis followed in frequency. The nature of the reaction was recorded as unknown if the patient could not recall the specifics in the history or if the chart was incomplete. The medical records revealed that the attending allergist determined the history alone was suggestive of an IgE mediated food allergy in a minority (37.3 %) of the patients and either not suggestive of food allergy or not clear enough to discern in the majority of patients (62.7 %). The patients who underwent food challenges generally had testing results that were discordant from their clinical history. A total of nine oral challenges were performed; almost all (7/9) had history suggestive of food allergy. Of these patients with suggestive histories, 5/7 had negative SPT and sIgE to the allergen of concern. The diagnosis of an IgE-mediated food allergy was made by the attending allergist in 77 (29.6 %) of patients and refuted in 126 (48.5 %). There was a sizeable minority of patients (n = 57; 21.9 %) in which the diagnosis remained unclear. Reasons for the ambiguity of diagnosis included active patients who still required follow up testing or incomplete chart.

**Table 2:** Food reaction characteristics and food allergy diagnosis rate in patient population

Characteristic	N (%)
Nature of reaction	
Cutaneous/angioedema	120 (46.2 %)
Gastrointestinal	47 (18.1 %)
Unknown	38 (14.6 %)
Anaphylaxis	29 (11.2 %)
Other/non specific	14.2 (5.4 %)
Respiratory	9 (3.5 %)
None	3 (1.2 %)
History suggestive of food allergy	
Yes	97 (37.3 %)
No/unclear	163 (62.7 %)
Positive	2 (22.2 %)
Negative	7 (77.8 %)
Diagnosis of food allergy	
Yes	77 (29.6 %)
No	126 (48.5 %)
Unclear	57 (21.9 %)

Table 3 details SPT and sIgE results for most common individual food allergens tested. The most frequently tested foods were peanuts and treenuts, fish, shellfish, milk, soy, sesame, egg and wheat. The majority of patients were tested to more than one allergen. Positive SPT and sIgE results were in the minority for all foods recorded. In particular, shellfish has the lowest rate of positive SPT and sIgE tests. Not all patients had both SPT and sIgE tests for a particular allergen. However, the kappa analysis for each allergen only included patients who had both SPT and sIgE to that allergen. Table 3 reflects the test results of patients who had both tests and were subsequently included in the kappa analysis.

**Table 3:** SPT and specific IgE results for Patients Included in Kappa Analysis

Food	Positive sIgE positive SPT	Negative sIgE negative SPT	Positive sIgE negative SPT	Negative sIgE positive SPT	Total
Peanut	26	41	16	4	87
Walnut	8	33	11	2	54
Hazelnut	11	19	28	2	60
Almond	3	35	21	1	60
Pistachio	6	4	1	1	12
Cashew	10	8	0	1	19
Pecan	5	26	5	1	37
Salmon	12	19	2	0	33
Codfish	12	21	2	2	37
Shrimp	9	32	6	1	48
Lobster	4	30	4	1	39
Crab	1	19	9	1	30
Clam	0	16	1	1	18
Milk	2	16	8	3	29
Soy	0	6	2	3	11
Sesame	6	2	2	0	10
Egg white	6	14	2	2	24
Wheat	1	16	8	0	25

**Fig. 1:**
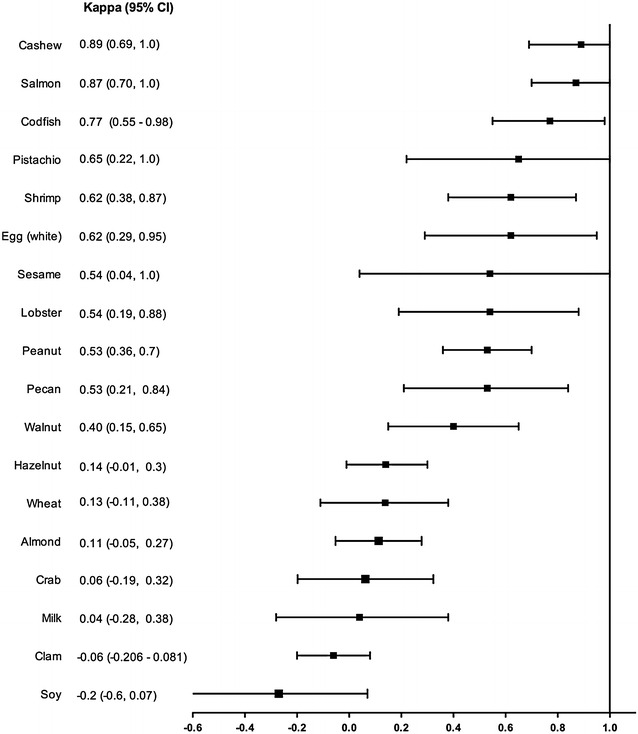
Kappa values

Figure 1 illustrates the kappa agreements coefficients for individual foods. SPT and sIgE agreement for cashew and salmon were near perfect. Agreement was substantial for pistachio, shrimp, and egg white, moderate for peanut, walnut, pecan, lobster, sesame, slight for hazelnut, almond, crab, milk, wheat, poor for soy and clam. There were not enough data points to perform kappa analysis on tuna, halibut, codfish, oyster, scallop, mussel and whole egg.

The kappa analyses reached significance (p < 0.05) to reject the null hypothesis for peanut, walnut, pistachio, cashew, pecan, salmon, shrimp, lobster and egg white. All of these kappa values showed at moderate or better agreement between the SPT and sIgE.

## Discussion

Our study shows that in adult patients being evaluated for a possible food allergy, agreement between the SPT and sIgE of common food allergens is at least moderate or better. Of the analyses that reached statistical significance, SPT and sIgE results showed near perfect agreement for cashew and salmon; substantial agreement for pistachio, codfish, shimp and egg white; moderate agreement for peanut, walnut, pecan, lobster. The value of objective testing in the diagnosis of IgE mediated food allergy is belied by the fact that the history of the reaction was frequently unclear and therefore not helpful. In this population, there appears to be substantial barriers to performing oral food challenges as very few were done. Our data did not supply any particular reason for the low number of oral challenges performed at our site. In general, both patients and physicians contribute to the reluctance to perform oral challenge. While clinicians often have financial or logistical barriers, patients generally contend with fear of experiencing a reaction or the significant time a challenge requires.

In our analysis, a significant p value (p < 0.05) rejects the null hypothesis that the kappa analysis result was due to chance. Peanut, walnut, pistachio, cashew, pecan, salmon, shrimp, lobster and egg white analyses reached a significant p value. Hazelnut, almond, crab, clam, milk, soy, wheat, sesame did not reach a significant p value probably because of small sample sizes and therefore their kappa analyses cannot be interpreted.

There is a paucity of literature examining the concordance of SPT and sIgE in adult populations. Asha’ari et al. showed that when tested for a predetermined panel of food allergens including peanut, egg, flour and chicken without clinical correlation, the agreement of SPT and sIgE was between fair to good [5]. This is comparable to our study in that there were no instances of poor agreement. Our data demonstrated a relatively stronger agreement between the SPT and sIgE to common food allergens. This may be due to the fact that the study population was tested for foods to which there was a history of symptoms after ingestion. Given the targeted approach to selecting food allergens to test for, our study population was less likely to produce discordant SPT and sIgE results due to asymptomatic sensitization.

The major strength of this study is that it is reflective of the routine clinical practice. Our study population was specific to patients in whom food allergy was already suspected because a reaction had occurred: highly representative of daily practice. The food allergens they were tested for at our allergy clinic reflected their clinical history or concern, making the results clinically relevant.

There are several limitations to our study. Inevitably, there was some heterogeneity to the data because this was a retrospective analysis; we could only abstract the data available on the medical records. We could also not obtain oral food challenges to validate the diagnosis of food allergy. We did not aim to correlate the agreement or the individual test results with an OFC validated diagnosis and it is not possible to do so from our data. Additionally, not every patient underwent both SPT and sIgE testing to the same panel of allergens because the referring physician had frequently ordered a broad panel of sIgE titres and at our allergy clinic a smaller selection of foods were skin prick tested. This reduced the number of patients who could be included in the kappa analysis and subsequent statistical analysis of several food allergens was not possible due to the limited sample size.

The literature in pediatric food allergy has established the utility of SPT and sIgE in predicting the result of oral food challenges and therefore their use in diagnosing food allergy. SPT wheal size and sIgE titre cutoffs that predict oral food challenge response have been characterized in children. This is so for peanut, fish, egg and milk while wheat and soy remain a challenge [9–12]. This advancement has likely led to greater roles of SPT and sIgE in diagnosing food allergy in children. In adults however, the utility and validity of sIgE is less well studied despite its frequent use. So far, there are no validated SPT or sIgE values that can predict a reaction on oral challenge test in adults as there are in children. It is further unclear whether the predictive values from the pediatric population can carry over to the adult population. The objective of our study was not to identify such cut offs, but the identification of these threshold values should be the direction of future research. The strength of the agreement between SPT and sIgE for common food allergens demonstrated by our study represents the first step of characterizing the utility of these tests in adults with suspected food allergy.

The data from this study may serve to reassure clinicians when both testing modalities are not available concurrently that the results will likely agree and that these two tests are possibly interchangeable. It may be useful for non-allergists who evaluate patients with complaints suggestive of IgE mediated food allergy to obtain sIgE to the foods of concern. Therefore when a patients presents with a history strongly suggestive of IgE mediated food allergy and a positive sIgE to the food of concern, referring physicians will be in a more confident position to triage the patient to specialist care or to counsel the patient appropriately while waiting for a specialist’s evaluation.

Our study also found a substantial minority of patients in whom a diagnosis of food allergy is still unclear after a thorough history and SPT or sIgE testing while the number of food challenges performed remained low. This finding should encourage allergists to use the oral challenge where appropriate and the provincial health services to remove any logistical or incentive barriers that discourage their use.

## Conclusion

In adults presenting with a concern of food allergy, the history alone cannot provide enough information to suggest the presence or absence of IgE mediated food allergy the majority of the time. Skin prick test and sIgE agree at least moderately well or better for peanut, walnut, pistachio, cashew, pecan, salmon, shrimp, lobster and egg white. These results may provide reassurance to clinicians when only one testing modality is available that the SPT and sIgE have a reliable degree of agreement.

## Abbreviations

SPT: skin prick test
Specific IgE: specific immunoglobulin E
